# Preliminary Incidence and Trends of Infections Caused by Pathogens Transmitted Commonly Through Food — Foodborne Diseases Active Surveillance Network, 10 U.S. Sites, 2016–2021

**DOI:** 10.15585/mmwr.mm7140a2

**Published:** 2022-10-07

**Authors:** Jennifer P. Collins, Hazel J. Shah, Daniel Lowell Weller, Logan C. Ray, Kirk Smith, Suzanne McGuire, Rosalie T. Trevejo, Rachel H. Jervis, Duc J. Vugia, Tamara Rissman, Katie N. Garman, Sarah Lathrop, Bethany LaClair, Michelle M. Boyle, Stic Harris, Joanna Zablotsky Kufel, Robert V. Tauxe, Beau B. Bruce, Erica Billig Rose, Patricia M. Griffin, Daniel C. Payne

**Affiliations:** ^1^Division of Foodborne, Waterborne, and Environmental Diseases, National Center for Emerging and Zoonotic Infectious Diseases, CDC; ^2^Minnesota Department of Health; ^3^New York State Department of Health; ^4^Oregon Health Authority; ^5^Colorado Department of Public Health and Environment; ^6^California Department of Public Health; ^7^Connecticut Emerging Infections Program; ^8^Tennessee Department of Health; ^9^University of New Mexico, Albuquerque, New Mexico; ^10^Georgia Department of Public Health; ^11^Maryland Department of Health; ^12^Center for Food Safety and Applied Nutrition, Food and Drug Administration, Silver Spring, Maryland; ^13^Food Safety and Inspection Service, U.S. Department of Agriculture, Washington, D.C.

To evaluate progress toward prevention of enteric infections in the United States, the Foodborne Diseases Active Surveillance Network (FoodNet) conducts active population-based surveillance for laboratory-diagnosed infections caused by *Campylobacter*,* Cyclospora*,* Listeria*,* Salmonella*, Shiga toxin-producing *Escherichia coli* (STEC), *Shigella, Vibrio*, and *Yersinia* at 10 U.S. sites. This report summarizes preliminary 2021 data and describes changes in annual incidence compared with the average annual incidence for 2016–2018, the reference period for the U.S. Department of Health and Human Services’ (HHS) Healthy People 2030 goals for some pathogens (*1*). During 2021, the incidence of infections caused by *Salmonella* decreased, incidence of infections caused by *Cyclospora*,* Yersinia*, and *Vibrio* increased, and incidence of infections caused by other pathogens did not change. As in 2020, behavioral modifications and public health interventions implemented to control the COVID-19 pandemic might have decreased transmission of enteric infections (*2*). Other factors (e.g., increased use of telemedicine and continued increase in use of culture-independent diagnostic tests [CIDTs]) might have altered their detection or reporting (*2*). Much work remains to achieve HHS Healthy People 2030 goals, particularly for *Salmonella* infections, which are frequently attributed to poultry products and produce, and *Campylobacter* infections, which are frequently attributed to chicken products (*3*).

FoodNet is a collaboration among CDC, 10 state health departments, the U.S. Department of Agriculture’s Food Safety and Inspection Service (USDA-FSIS), and the Food and Drug Administration (FDA). FoodNet’s catchment area (Connecticut, Georgia, Maryland, Minnesota, New Mexico, Oregon, Tennessee, and selected counties in California, Colorado, and New York) includes approximately 15% of the U.S. population (an estimated 50 million persons in 2020). Bacterial infections were diagnosed by culture or CIDT; *Cyclospora* infections were diagnosed by microscopy or polymerase chain reaction (*2*). The frequencies of hospitalizations,* deaths,^†^ outbreak-associated infections,^§^ and international travel–associated infections^¶^ were calculated overall and by pathogen; unknown results were classified as “no.” Incidence was calculated by dividing the number of laboratory-diagnosed infections in 2021 by 2020 U.S. Census Bureau population estimates for the surveillance area. The percentage change in incidence during 2021 compared with the average annual incidence during 2016–2018 was estimated using a new Bayesian, negative binomial model with penalized thin plate splines that adjusted for state-specific trends and changes in population over time (*4*).

Surveillance for physician-diagnosed postdiarrheal hemolytic uremic syndrome (HUS), a complication of STEC infection, is conducted through a network of nephrologists and infection preventionists and by hospital discharge data review. This report includes HUS cases in children and adolescents aged <18 years for 2020, the most recent year with available data. This activity was reviewed by CDC and was conducted consistent with applicable federal law and CDC policy.**

During 2021, FoodNet identified 22,019 infections, 5,359 hospitalizations, and 153 deaths (Table 1). Incidence was highest for *Campylobacter* (17.8 cases per 100,000 population) and *Salmonella* (14.2). Overall, 8% fewer infections were reported during 2021 than the average during 2016–2018; incidence decreased for *Salmonella,* increased for *Cyclospora, Vibrio*, and *Yersinia,* and was unchanged for *Campylobacter, Listeria*, *Shigella,* and STEC. The percentage of infections resulting in hospitalization and the percentage of outbreak-associated infections were stable. Overall, 7% of infections in 2021 were associated with international travel compared with 13% during 2016–2018 (Figure).

**TABLE 1:** Number of laboratory-diagnosed bacterial and parasitic infections, hospitalizations, deaths, outbreak-associated infections, crude incidence, and percentage change compared with 2016–2018 average annual incidence, by pathogen — Foodborne Diseases Active Surveillance Network, 10 U.S. sites,* 2021^†^

Pathogen	2021					% Change in infection incidence (95% CrI^¶¶^), 2016–2018 to 2021
	No. of Infections^§^	No. (%)			Crude incidence^§§^	
		Hospitalizations^¶^	Deaths**	Outbreak-associated infections^††^		
**Total**	**22,019**	**5,359 (24)**	**153 (0.7)**	**861 (4)**	**—**	**—**
**Bacteria**						
*Campylobacter*	8,974	1,822 (20)	33 (0.4)	51 (0.6)	17.8	−5.5 (−11.4 to 0.9)
*Salmonella*	7,148	1,974 (28)	52 (0.7)	597 (8)	14.2	−10.0 (−16.9 to −3.2)
STEC***	2,542	600 (24)	10 (0.4)	79 (3)	5.0	8.8 (−6.8 to 27.0)
*Shigella*	1,699	532 (31)	8 (0.5)	67 (4)	3.4	−14.8 (−33.8 to 6.0)
*Yersinia*	683	146 (21)	3 (0.4)	2 (0.3)	1.4	79.0 (49.4 to 116.1)
*Vibrio*	461	117 (25)	9 (2)	8 (2)	0.9	45.5 (26.9 to 66.3)
*Listeria*	148	140 (95)	37 (25)	9 (6)	0.3	4.6 (−8.5 to 20.1)
**Parasite**						
*Cyclospora*	364	28 (8)	1 (0.3)	48 (13)	0.7	443.2 (195.9 to 1,134.2)

**Abbreviations:** CIDT = culture-independent diagnostic test; CrI = credible interval; STEC = Shiga toxin–producing *Escherichia coli*.

* Data were obtained from laboratories in Connecticut, Georgia, Maryland, Minnesota, New Mexico, Oregon, Tennessee, and selected counties in California, Colorado, and New York.

^†^ 2021 data are preliminary.

^§^ Bacterial infections diagnosed by culture or CIDT. *Cyclospora* infections diagnosed by microcopy or polymerase chain reaction.

^¶^ Admission to an inpatient unit or an observation stay of >24 hours within 7 days before or after specimen collection or determined to be related to the infection if beyond this time frame. Absolute change in percentage of infections resulting in hospitalization during 2021 compared with annual average for 2016–2018: *Campylobacter* (0.3), *Salmonella* (0.3), STEC (1), *Shigella* (8), *Yersinia* (−4), *Vibrio* (−5), *Listeria* (−2), *Cyclospora* (2), and overall (0.6). Unknown hospitalization status (10% of infections during 2021 and 4% during 2016–2018) was classified as not hospitalized.

** Attributed to infection when deaths occurred during hospitalization or within 7 days after specimen collection for nonhospitalized patients. Absolute change in percentage of infections resulting in death during 2021 compared with annual average for 2016–2018: *Campylobacter* (<0.1), *Salmonella* (0.3), STEC (<0.1), *Shigella* (0.4), *Yersinia* (−0.7), *Vibrio* (−0.2), *Listeria* (6), *Cyclospora* (0.1), and overall (0.2). Unknown death status (8% of infections during 2021 and 3% during 2016–2018) was not classified as a death.

^††^ Generally defined as two or more cases of similar illness associated with a common exposure; some sites also stipulate that illnesses be from more than one household. Absolute change in percentage of outbreak-associated infections during 2021 compared with annual average for 2016–2018: *Campylobacter* (0.2) *Salmonella* (1), STEC (−1), *Shigella* (−1), *Yersinia* (0.2), *Vibrio* (−2), *Listeria* (1), *Cyclospora* (−10), and overall (<0.1). Unknown outbreak-association status (0.02% of infections during 2021 and 0% during 2016–2018) was classified as not outbreak-associated.

^§§^ Cases per 100,000 population. Domestic incidences (cases with no or unknown travel) by pathogen during 2021: *Campylobacter* (17.0), *Salmonella* (13.1), STEC (4.6), *Shigella* (3.0), *Yersinia* (1.3), *Vibrio* (0.8), *Listeria* (0.3), and *Cyclospora* (0.6).

^¶¶^ Percentage change reported as increase or decrease. Some increases are likely due to increasing use of CIDTs by clinical laboratories.

*** Compared with the annual average for 2016–2018, the incidence of STEC O157 infections (0.6 per 100,000) changed by −21.7% (95% CrI = −32.4% to −11.5%), and the incidence of non-O157 STEC infections (1.8) changed by −11.6% (95% CrI = −26.2% to 7.0%).

**FIGURE:**
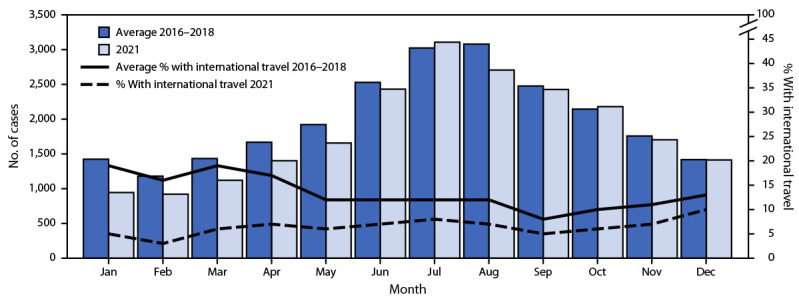
Number of laboratory-diagnosed bacterial and parasitic infections and percentage of persons with international travel,* by month — Foodborne Diseases Active Surveillance Network, 10 U.S. sites,^†^ 2016–2018 and 2021^§^ * History of international travel before illness began: 30 days for *Listeria* and *Salmonella* serotypes Typhi and Paratyphi, 14 days for *Cyclospora*, and 7 days for other pathogens. Unknown international travel (25% of infections during 2021 and 17% during 2016–2018) was classified as no travel. ^†^ Data were obtained from laboratories in Connecticut, Georgia, Maryland, Minnesota, New Mexico, Oregon, Tennessee, and selected counties in California, Colorado, and New York. ^§^ 2021 data are preliminary.

Two thirds (67%) of bacterial infections were diagnosed using CIDT in 2021, compared with approximately one half (49%) during 2016–2018 (Table 2). In 2021, 37% of bacterial infections were diagnosed using only CIDT (i.e., the specimen had a negative culture result or was not cultured) compared with 26% during 2016−2018. A reflex culture^††^ was performed for 70% of infections diagnosed by CIDT in 2021, similar to 2016–2018. Reflex culture attempts decreased for *Campylobacter, Listeria,* STEC, *Vibrio*, and *Yersinia*. The percentage of reflex cultures that yielded a pathogen ranged from 24% for *Yersinia* to 89% for *Listeria*.

**TABLE 2:** Percentage of bacterial infections diagnosed by a culture-independent diagnostic test, only by a culture-independent diagnostic test, with a reflex culture, and percentage of reflex cultures that yielded a pathogen — Foodborne Diseases Active Surveillance Network, 10 U.S. sites,* 2016–2018 and 2021^†^

Pathogen		Infections diagnosed by CIDT, %^§^		Infections diagnosed only by CIDT, %^¶^		Infections with a reflex culture, %**		Reflex culture yielded a pathogen, %^††^	
		2016–2018	2021	2016–2018	2021	2016–2018	2021	2016–2018	2021
**Overall**	**49**		**67**	**26**	**37**	**71**	**70**	**65**	**64**
*Campylobacter*	53		70	36	46	60	56	55	62
*Listeria*	4		13	0	2	100	95	88	89
*Salmonella*	30		49	9	15	79	85	88	83
*Shigella*	49		76	29	44	69	83	58	51
STEC	100		100	43	53	88	80	65	59
*Vibrio*	45		61	31	46	83	73	38	33
*Yersinia*	69		85	46	71	69	68	48	24

**Abbreviations:** CIDT = culture-independent diagnostic test; STEC = Shiga toxin–producing *Escherichia coli*.

* Data were obtained from laboratories in Connecticut, Georgia, Maryland, Minnesota, New Mexico, Oregon, Tennessee, and selected counties in California, Colorado, and New York.

^†^ 2021 data are preliminary.

^§^ Includes specimens that had a culture performed, regardless of the result, and those not cultured. Denominator is total infections.

^¶^ Includes specimens that had a negative culture result and those not cultured. Denominator is total infections.

** Specimens with a positive CIDT result that had a culture performed, regardless of the result. Denominator is infections diagnosed by CIDT.

^††^ Denominator is specimens with a reflex culture.

Among 6,110 *Salmonella* isolates, 5,442 (89%) were serotyped in 2021. The seven most common serotypes were Enteritidis (908; 17%), Newport (596; 11%), Typhimurium (510; 9%), Javiana (406; 7%), I 4,[5],12:i:- (304; 6%), Oranienburg (247; 5%), and Infantis (232; 4%). Compared with 2016–2018, incidence^§§^ was higher for Oranienburg (38.6% increase; 95% credible interval [CrI] = 14.2% to 72.1%) and Infantis (23.7%; 95% CrI = 2.9% to 48.7%), lower for I 4,[5],12:i:- (−33.4%; 95% CrI = −45.4% to −17.9%), Typhimurium (−29.2%; 95% CrI = −35.7% to −22.4%), and Enteritidis (−24.7%; 95% CrI = −33.6% to −15.6%), and unchanged for Javiana (−23.0%; 95% CrI = −44.0% to 12.4%) and Newport (−8.7%; 95% CrI = −28.5% to 19.2%). Enteritidis, Newport, Typhimurium, Javiana, and I 4,[5],12:i:- have been among the five most common serotypes since 2010. Infantis has been among the 10 most common since 2013. During 2021, Oranienburg caused a multistate outbreak linked to onions;^¶¶^ before that, Oranienburg had last been among the 10 most common serotypes in 2009.

Among 1,203 STEC isolates in 2021, serogroup O157 was most common (314; 26%), followed by O26 (179; 15%), O103 (140; 12%), and O111 (116; 10%). During 2020, FoodNet identified 49 cases of postdiarrheal HUS in children and adolescents aged <18 years (0.4 cases per 100,000), including 21 (43%) in children aged <5 years (0.7 per 100,000). The overall incidence of HUS was similar to that during 2016–2018 (−7.6% change; 95% CrI = −21.1% to 8.4%). The 2020 incidence of STEC O157 infections decreased 16.8% (95% CrI = −25.0% to −9.3%) compared with the average during 2016–2018. Overall, 37 (76%) HUS cases had evidence of STEC infection; 18 of 23 (78%) HUS cases with culture-confirmed STEC infection were serogroup O157.

## Discussion

The 8% decrease in enteric infections reported to FoodNet during 2021 compared with the annual average during 2016–2018 suggests ongoing effects of the COVID-19 pandemic. Previously published FoodNet data (*2*) and other studies using data from 2020 (*5*–*7*) support the occurrence of two pandemic-related phenomena: decreased transmission and incidence of enteric infections (i.e., due to pandemic control measures) and underascertainment of infections related to changes in health care–seeking behaviors (e.g., increased use of telemedicine). The relatively low percentage of infections associated with international travel during 2021 (7%) and 2020 (5%) (*2*) support occurrence of the former. Lifting of pandemic control measures might have contributed to the stable or increased incidence for some pathogens during 2021. The stable percentage of hospitalizations during 2021 suggests that underascertainment was similar to baseline levels. However, the stable incidence of HUS coincident with a decrease in incidence of STEC O157 infections during 2020 suggests that these infections and perhaps others were underascertained; the severity of HUS makes it a more reliable measure (i.e., less affected by changes in health care delivery or health care–seeking behaviors). A better understanding of how pandemic control measures influenced enteric infections might help identify interventions to sustainably decrease their incidence.

Increasing use of CIDTs complicates the interpretation of surveillance trends, with factors such as test platform and pathogen affecting the accuracy of results. Molecular tests have high sensitivity for many pathogens (*8*) but might not indicate viable organisms. Variable specificity of CIDTs for FoodNet pathogens can result in false-positive results, most notably for *Vibrio* (*9*). Reflex cultures remain essential for public health functions, including determining antibiotic resistance, detecting outbreaks, and determining serotypes.

Comprehensive efforts are needed to address the root causes of foodborne illness, and substantial progress is needed to achieve HHS Healthy People 2030 goals, particularly for *Salmonella* and *Campylobacter* (*1*). The most recent report from the Interagency Food Safety Analytics Collaboration attributed 23% of foodborne *Salmonella* illnesses to chicken and turkey and 42% to produce items (*3*). The predominance of five *Salmonella* serotypes for >10 years emphasizes the need for more robust measures to identify and address *Salmonella* contamination in food by serotype. In October 2021, USDA-FSIS announced plans for stronger efforts to reduce *Salmonella* infections associated with poultry products, including before harvest and in slaughter and processing facilities, and began working with a national advisory committee*** (*10*). Targeted efforts are also needed to address *Salmonella* contamination of produce and *Campylobacter* infections from chicken products (*3*). Improving agricultural water safety, as FDA has proposed,^†††^ might decrease infections with pathogens transmitted commonly by produce, including *Salmonella*, STEC O157, and *Listeria*.

The findings in this report are subject to at least three limitations. First, infections resulting from all modes of transmission (i.e., not exclusively foodborne) are included. Second, changes in incidence might not reflect sustained trends, particularly in the context of the COVID-19 pandemic. Finally, the percentage of cases with hospitalization, death, and international travel might be underestimated because unknown results were classified as “no”; preliminary 2021 data have a higher percentage of unknown results than do finalized 2016–2018 data.

FoodNet’s 2021 data demonstrate ongoing effects of the COVID-19 pandemic on reported cases of infections transmitted commonly through food. As CIDT use continues to increase, reflex cultures remain essential for public health functions. Identifying novel strategies and implementing known strategies to address the root causes of illness are needed to sustainably decrease infections and achieve HHS Healthy People 2030 goals.

**Summary**

**What is already known about this topic?**

During 2020, the number of infections reported to the Foodborne Diseases Active Surveillance Network (FoodNet) decreased compared with the average reported during 2016–2018. Pandemic-related measures likely decreased occurrence of some infections and limited ascertainment of others.

**What is added by this report?**

During 2021, the number of infections reported to FoodNet decreased 8% compared with the 2016–2018 average, likely related to the pandemic. Most infections were caused by *Campylobacter* or *Salmonella;* the five most common *Salmonella* serotypes remained predominant. Use of culture-independent diagnostic tests increased.

**What are the implications for public health practice?**

Comprehensive efforts are needed to improve food safety. Substantial progress is needed to achieve national goals, particularly for *Salmonella* and *Campylobacter*. Reflex cultures remain essential for surveillance of enteric infections.

## Footnotes

* Admission to an inpatient unit or an observation stay of >24 hours within 7 days before or after specimen collection or determined to be related to the infection if beyond this time frame.

† Attributed to infection when they occurred during hospitalization or within 7 days after specimen collection for nonhospitalized patients.

§ Generally defined as two or more cases of similar illness associated with a common exposure; some sites also stipulate that illnesses be from more than one household.

¶ International travel before illness began: 30 days for *Listeria* and *Salmonella* serotypes Typhi and Paratyphi, 14 days for *Cyclospora,* and 7 days for other pathogens.

** 45 C.F.R. part 46. 102(I)(2), 21 C.F.R. part 56; 42 U.S.C. Sect. 241(d); 5 U.S.C. Sect. 552a; 44 U.S.C. Sect. 3501 et seq.

†† Culture of a specimen with a positive CIDT result.

§§ 2021 incidence (per 100,000): Enteritidis (1.8), Newport (1.2), Typhimurium (1.0), Javiana (0.8), I 4,[5],12:i:- (0.6), Oranienburg (0.5), and Infantis (0.5).

¶¶
https://www.cdc.gov/salmonella/oranienburg-09-21/details.html

*** The National Advisory Committee on Microbiological Criteria for Foods.

†††
https://www.fda.gov/food/food-safety-modernization-act-fsma/fsma-proposed-rule-agricultural-water
